# ‘Having to learn this so late in our lives…’ Swedish elderly patients’ beliefs, experiences, attitudes and expectations of e-health in primary health care

**DOI:** 10.1080/02813432.2019.1570612

**Published:** 2019-02-08

**Authors:** Veronica Milos Nymberg, Beata Borgström Bolmsjö, Moa Wolff, Susanna Calling, Sofia Gerward, Magnus Sandberg

**Affiliations:** a Department of Clinical Sciences, Lund University, Malmö, Sweden;; b Center for primary Health Care research Malmö, Malmö, Sweden;; c Innovation Skåne, Lund, Sweden;; d Department of Health Sciences, Lund University, Lund, Sweden

**Keywords:** E-health, elderly, primary health care, focus groups, qualitative research

## Abstract

**Objective:** The elderly are an increasing group and large consumers of care in Sweden. Development of mobile information technology shows promising results of interventions for prevention and treatment of chronic diseases. Exploring the elderly patients’ beliefs, attitudes, experiences and expectations of e-health services helps us understand the factors that influence adherence to such tools in primary care.

**Material and methods:** We conducted focus group interviews with 15 patients from three primary health care centers (PHCCs) in Southern Sweden. Data were analysed with thematic content analysis with codes and categories emerged from data during analysis.

**Results:** We found one comprehensive theme: ‘The elderly’s ambivalence towards e-health:  reluctant curiosity, a wish to join and need for information and learning support’. Eight categories emerged from the text during analysis: ‘E-health – a solution for a non-existing problem?’, ‘The elderly’s experiences of e-health’, ‘Lack of will, skills, self-trust or mistrust in the new technology’, ‘Organizational barriers’, ‘Wanting and needing to move forward’, ‘Concerns to be addressed for making e-health a good solution’, ‘Potential advantages with e-health versus ordinary health care’ and ‘Need for speed, access and correct comprehensive information’.

**Conclusions:** Elderly patients in Sweden described feelings of ambivalence towards e-health, raising concerns as accessibility to health care, mistrust in poor IT systems or impaired abilities to cope with technology. They also expressed a wish and need to move forward albeit with reluctant curiosity. Successful implementation of e-health interventions should be tailored to target different attitudes and needs with a strong focus on information and support for the elderly.

**Key points**
- Exploring the elderly patients’ beliefs, experiences, attitudes and expectations of the fast developing e-health services helps us understand the factors that influence adherence to such tools in primary care.
- Elderly patients in Sweden reported ambivalence and different experiences and attitudes towards e-health, raising concerns as accessibility to health care, costs and mistrust in poor IT systems or impaired abilities to cope with technology.
- They also expressed a wish and need to move forward albeit with reluctant curiosity. Successful implementation of e-health interventions should be tailored to target different attitudes and needs with a strong focus on information and support for the elderly.

## Introduction

The future demographic changes with an aging population are one of the most important challenges for policy makers and authorities in Sweden, as well as in other Western countries [1]. An increasing life expectancy and positive trends of self-rated health also indicate that elderly people in Sweden keep themselves alert and vital through high access to medical advances, healthy diet and exercise [2]. A better informed and healthier generation of elderly people will likely demand better access to health care [3]. The era of mobile information technology opens new possibilities, with implementation of a large variety of mobile solutions in health care; methods described in literature as ‘e-health’ or ‘tele-health’. Previous studies have shown promising results with evidence that non-interactive or interactive interventions such as sms-texting and reminders significantly improve compliance to medications, follow-up rate or disease monitoring [4–7]. A recent systematic review concluded that the use of mobile phone technology to support self-monitoring in long-term conditions such as chronic obstructive pulmonary disease (COPD), hypertension and heart failure can lead to significant reductions in hospitalizations and readmissions to hospitals [8]. A meta-analysis of telehealth programs for patients with chronic heart failure demonstrated better clinical effectiveness compared to usual care [9]. In a systematic review, patient quality of life and satisfaction using telehealth services for monitoring of heart failure was found to be similar or better compared to usual care [10].

Primary health care in Sweden is managing the first-line health care offering both prevention, monitoring and treatment for a majority of chronical diseases. Aging is known to be associated with an increased prevalence of multiple chronical diseases thus making the elderly a special group of patients in need of special attention. Given the increasing evidence concerning the value of digital tools in monitoring of chronical conditions [4–8], development of e-health care services for the elderly population should be a prioritized area. One of the big challenges in Swedish healthcare is the diversity of different electronic medical records (EMRs) in hospital care, primary care and community provided elderly care; such challenges can threaten patient safety [11]. Although efforts are made by the policy makers to create a common IT-platform, information about update of treatment and medication lists often gets lost between healthcare providers and can put patients’ safety at risk [12].

Meanwhile, the constant and fast development of digital tools is a challenge for both health care providers, policy makers and patients, due to the diversity of attitudes and behaviors among patients towards new technologies. Several theories and models have attempted to explain the concepts of accepting and adopting novel technologies and behaviors [13,14]. One of them, Rogers’ diffusion of innovations theory, offers a conceptual framework for understanding the complex process of adopting novel technologies and behaviours [14]. Rogers describes five factors that may influence the rate of adoption: (1) the adopter’s perception of the relative advantage of the innovation; (2) the compatibility of the innovation with existing structures; (3) the perceived degree of difficulty involved in adopting the innovation; (4) the testability of the innovation, in the absence of significant resources; and (5) the visibility of outcomes resulting from adoption of the innovation [14]. In accordance with this theory, a previous study showed that attitudes towards e-health among the elderly might diverge with favorable attitudes being associated with higher education level or with having a computer and that elderly people select such services even if they are not overly enthusiastic about using digital tools [15]. The aim of this study is to explore elderly patients’ beliefs, attitudes, experiences and expectations in order to understand the factors influencing their adherence to new innovative e-health instruments.

## Material and methods

### Design

The study design has a qualitative approach using focus group interviews for data collection [16].

### Settings

Patients were strategically recruited from three primary health care centers (PHCCs) in Southern Sweden from different socioeconomic areas. Three of the authors worked as GPs at the three included PHCCs (SC at PHCC no 1, MW at PHCC no 2, BBB at PHCC no 3) and were involved in recruiting patients between Sept-Dec 2016.

### Participants

Elderly patients (65–80 years) were included in the study. Inclusion criteria were patients with at least one chronic disease (hypertension, diabetes, COPD), treatment with at least three drugs continuously and no previous diagnose of cognitive impairment. Eligible patients were identified by physicians at the PHCCs. All eligible patients, who came to a planned appointment at the PHCC and who met the inclusion criteria, were invited to participate at the start of Sept 2016 with the goal to reach six participants in each focus group. They received an invitation letter which contained information about the study such as that the study was voluntary and that there was the possibility to withdraw at any time without affecting their contacts with the healthcare organizations. Data were not collected about the reason for not participating. Interviews were performed between Jan-March 2017 at PHCC no 1, 2 and 3 consecutively.

### Data collection and analysis

The interviews were performed at the PHCC where the patients were registered. Patients received no compensation for participating in the interviews. However, coffee and fruit were offered to the participants. Focus group interviews were conducted using a semi-structured interview guide. Interview questions were created with an emphasis on the following themes: attitudes and beliefs about e-health services, expectations of such tools, motivational factors and barriers. The interviews were conducted by the first author of the study (VMN), a GP with prior experience of leading focus group interviews. Different assistants for every focus group (BBB for PHCC no 1, SC and SG for PHCC no 2 and MW for PHCC no 3) took notes in order to recall non-verbal communication within the group during the interview. To encourage the participants to have a broad view of e-health, the interviews were started with the moderator (VMN) reading aloud a hypothetical case of an older female patient who gets palpitations sitting at home, becomes concerned and makes contact with her PHCC online in order to get help. In the hypothetical case, she is able to record an ECG through her mobile phone, sends it to the nurse who reassures the patient of the normal ECG and offers contact with the GP the following day. The participants were later invited to share their thoughts about the story and discuss their own experiences of mobile phones as a digital tool with access to internet (smart-phones), seeking medical information online, contacting the health care system online or self-monitoring of chronic illnesses. Directly after the interviews, participants were asked to fill in a form with questions about their living conditions, educational level, sex, age and use of digital tools (internet, e-mail and smart-phone habits).

The interviews were audio recorded, transcribed verbatim and studied using thematic content analysis [17,18]. After reading the transcribed interviews and additional notes gathering non-verbal information, the text was divided into meaning units, condensed and then labelled with a code. The codes with similar content were compiled into different subcategories using an excel file and labelled with the interview number and page in order to secure the source of the meaning unit. The subcategories were grouped into categories, which in turn generated one comprehensive theme. Two of the researchers (VMN and MS) performed the initial data analysis with coding and compilation. The results were later discussed with the other researchers until consensus was reached. The thematic content analysis method was used with codes and categories derived from data analysis [16]. An example of the text condensation in meaning units is shown in Table 1. The dataset (in translation) is available on request from the authors.

**Table 1.** Example of text condensation and coding.

Meaning unit	Condensed meaning unit	Initial coding	Final coding (sub-category)	Category
‘I feel safe now, they call me for example every three months…and they check the blood sugar and everything…if it is all right I feel happy with it, I mean…it won´t turn into a disaster in three months, so I get this little check-up…’	I feel safe with regular visits every three months for my diabetes	Security with regular visits at the PHCC	Do not fix what is not broken	E-health-a solution for a non-existing problem?
‘In my case, having these chronic diseases…it demands that I come here once a year to see the doctor, and once more to see a nurse. So I haven't had any contact with this e-health yet.’	Having chronic illnesses demands doctor and nurse visits every year, buy I don’t have any experience of e-health yet.	Lack of experiences of digital tools	Lack of experiences or knowledge of digital tools	Elderly’s experiences of e-health
‘Because I hate everything with computers, I am almost allergic to them, so I don't want to see myself in that world’	I hate computers and I don´t want to use them	Aversion to technology	Lack of interest for digital tools and aversion to technology	Lack of will, skills, self-trust or mistrust in the new technology
‘I have understood, after following the debate many times, when it comes to health care, that they work in different systems in different places, for example hospitals and primary health care centers…without any connection so to speak…’	The health care system uses different IT systems without any connection to each other	Lack of communication between IT systems in health care	Poor communication between health care IT systems	Organizational barriers
‘I am not very good at this…but I think I am online maybe an hour a day…and google stuff, if it's not names it might be something else, and I have looked at medical pictures….But it is dangerous too… actually I don't sit and think that I might be ill with this and that…but you can still get some advice…’	I am often on line and google medical information. I do not imagine myself sick but I look for advice on line.	Positive with seeking medical information from the internet	Curiosity and interest for digital tools and technical solutions	Wanting and needing to move forward
‘The risk with putting so much (money) on applications and stuff like this, is that you might need to cut down on personnel a bit…and maybe shut down a health care center…and then more cut offs might come…as they believe that personnel is expensive…’	The risk with putting so much resources on technology is that it might lead to health care personnel cut offs	Concerns about use of resources and costs	Accessibility, costs and other risks with e-health	Concerns to be addressed for making e-health a good solution
‘It is an advantage if you can do it (online booking), because many times when you call, for example now, during the whole December…it´s been sixteen people before me on the telephone queue every time I´ve called. It´s been very disturbing…’	It is an advantage to be able to book online when it is a long queue on the telephone line.	Advantage with online booking as a complement to ordinary telephone booking	Online booking as a complement	Potential advantages with e-health versus ordinary health care
‘But maybe after a couple of months I need to get in contact with her (the GP), and the process gets bigger and longer, and I need to come down here and make an appointment…instead I could get in contact by phone, or maybe sending a photo with my phone…But usually I think it is nicer to have the physical contact.’	It is nicer to have physical visits but digital contact in between visits would get me more rapidly in contact with my GP.	Need for digital consultations	Need for digital consultations in certain situations	Need for speed, access and correct comprehensive information

## Ethical approval

The research team applied and received ethical approval to perform the study from the Regional Ethical Review Board in Lund, Dnr 2016/812.

## Results

A total of 24 patients were approached, 18 consented to participate (six patients from every PHCC) and 15 came to the interviews (6, 6 and 3). The age of the participants ranged from 65–80, eight women and seven men (Table 2).

**Table 2.** Patients’ characteristics.

Primary Health Care Center	Age	Sex	Education
PHCC no 1	73	female	Secondary school^a^
	78	female	Secondary school^a^
	67	male	High school^b^
	80	female	Secondary school^a^
	70	male	Secondary school^a^
	77	male	High school^b^
PHCC no 2	75	female	High school^b^
	76	male	Secondary school^a^
	72	male	Secondary school^a^
	75	female	University
	68	female	Secondary school^a^
	73	female	Secondary school^a^
PHCC no 3	80	male	University
	65	male	High school^b^
	66	female	Secondary school^a^

^a^Has completed 9 years of education.

^b^Has completed 12 years of education.

We found one comprehensive theme: ‘The elderly’s ambivalence towards e-health: reluctant curiosity, a wish to join and need for information and learning support’. During analysis, 36 subcategories emerged from the text and resulted in 8 different categories as shown in Table 3. The categories will be listed below with the subcategories embedded in the text with bold characters, some of the subcategories being exemplified with citations.

**Table 3.** Categories and subcategories.

Category	Subcategories
E-health – a solution for a non-existing problem?	Do not fix what is not broken Problems today that e-health might solve Importance of accessibility to physician regardless of contact way
Elderly’s experiences of e-health	Positive experience and knowledge about digital tools Lack of experiences and knowledge Unmet expectations of e-health Dislike of text messages for health monitoring and life style advices
Lack of will, skills, self-trust or mistrust in the new technology	Mistrust in knowledge and know how about technology in elderly Too high knowledge demands on elderly Insecurity and fear with technology in today’s system The ageing body as a barrier Lack of interest for digital tools and aversion to technology
Organizational barriers	Lack of IT competence in health care organizations Who is responsible when IT systems fail? Poor communication between health care organizations' IT systems Disappointment over poor IT systems Mistrust in e-health from health care organizations
Wanting and needing to move forward	Cannot stop development Curiosity and interest for digital tools and technical solutions Need for help and information concerning e-health To learn on older days
Concerns to be addressed for making e-health a good solution	Lack of triage with online booking Accessibility, costs, and other risks with e-health Lack of time for physicians despite e-health Insecurity with e-health in emergency situations
Potential advantages with e-health versus ordinary health care	Better access with video consultations Practical and safe with a comprehensive drug list in the mobile E-health a future way to reduce bureaucracy, demands and time Online booking as a complement Advantages of digital tools for some
Need for speed, access and correct comprehensive information	Expectations of higher accessibility with e-health Need for fast e-health accessibility in emergency situations Importance of trustworthy information online Expectations of lab results online Need for comprehensive drug list Need for digital consultation in certain situations

### *‘E-health – a solution for a non-existing problem?*’

This category emerged from three different subcategories, as shown in Table 3. In this category, the participants described that there was ‘**No need to *fix what wasn’t broken****’,* including experiences about how the health care system works today and their satisfaction with the present model with regular visits at the PHCC or telephone contacts with the nurse or the GP. Some of the interviewees described their preferable choice of receiving ordinary letters rather than electronic messages and conveyed a lack of interest for digital solutions. These participants also emphasized trust and safety with the usual visits and contact ways in contrast to the hypothetical video consultation scenario presented in the introduction part.

I feel safe now, they call me, for example, every three months…and they check the blood sugar and everything…if it is all right I feel happy with it, I mean…it won´t turn into a disaster within three months, so I get this little check-up… (Interview no 3, patient no 3)

However, not all participants experienced that the health system today worked smoothly. Some of the participants described irritation over the nurse as a gate keeper in the overloaded telephone triage system, and the need for higher accessibility to health care outside office hours. They experienced this as ‘**Problems today that e-health might solve**’, estimating e-health as a potential complement in the matter of accessibility to health care. They stressed ‘**The importance of accessibility to a physician regardless of contact way’**. However, a majority experienced high accessibility to the telephone triage and booking system and expressed trust in the nurses’ competence.

### ‘Elderly’s experiences of e-health’

Concerning e-health, the participants had different experiences and different levels of knowledge. Some of them had ‘**Positive experiences and knowledge about digital tools**’ not related to health, such as internet banking or using an electronic ID. A majority of patients expressed ‘**Lack of experiences and knowledge**’ of digital tools and only a few had tried to get in contact with the primary health care system online on the PHCC’s website. The participants with some experience of e-health talked about seeking medical information online, reading the electronic medical record (EMR) or ordering drugs from an internet pharmacy. Those who had some experience described poor features of the PHCC’s website with lack of functions or difficulties to access important information with ‘**Unmet expectations of e-health**’:

So I went in on PHCC’s web page and looked, and it was easy, but it wasn’t possible to get in contact with any doctor…it would be great, as you say, to make an appointment within two or three weeks, but it wasn't possible. And I looked further for March and April, I thought, maybe it is possible then, but it wasn't…so it doesn't work here anyway… (Interview no 1, person no 2)

One of the participants who was seeking medical information online on a daily basis expressed her ambivalence towards it as following:

I am not very good at this…but I think I am online maybe an hour a day and google stuff, if it's not names it might be something else, and I have looked at medical pictures….But it is dangerous too… actually I don't sit and think that I might be ill with this and that…but you can still get some advice… (Interview no 2, person no 1)

Patients with some experience of digital tools expressed interest in receiving text messages as reminders for self -monitoring of chronic disease or as reminders for appointments. They also had thoughts about differences between the organizations. One of the participants wondered why short text message reminders are common in the dental care but not in primary care. The patients with limited experiences of digital tools expressed ‘**Dislike of text messages for health monitoring and lifestyle advices**’ with some of them describing an additional risk of increased anxiety between the regular visits at the PHCC.

### ‘Lack of will, skills, self-trust or mistrust in the new technology

The elderly expressed ‘**Mistrust in knowledge and know-how in elderly**’ and mistrust in their own capabilities, even among those that had no experience in using digital tools. Some of the participants experienced ‘**Too high knowledge demands on elderly**’. They described a generation gap with younger generations being more accustomed to the new technology and the elderly’s own insecurity with the digital tools and difficulties to learn new things. They described ‘**Insecurity and fear with technology in today’s system**’ such as fear of being displaced in the booking system or not being able to manage access to health care in emergency situations. They also expressed stress caused by not being able to keep up with the knowledge needed to handle digital tools, and described ‘**The aging body as a barrier**’ with impaired practical abilities such as trembling fingers or impaired vision or hearing.

Having this age…we are in a kind of a shift period…having to learn this so late in our lives. The young ones, in their twenties, they don´t think as we do at all…It gets more natural eventually, but for our age group, the knowledge is worse, absolutely. And then, they (the young ones) see well, and hear well, when it (the phone) rings and so on…they have other abilities. (Interview no 2, person no 4)

One of the patients, with no experience of smart phone or internet use, expressed her ‘**Lack of interest for digital tools and aversion to technology**’ as following:

Because I hate everything with computers, I am almost allergic to them, so I don't want to see myself in that world… (Interview no 3, person no 2)

### ’Organizational barriers’

In this category patients described a clear and almost unanimous disappointment in health care using poor IT systems. This is expressed in the subcategories: ‘**Lack of IT competence in health care organizations’** and **‘Who is responsible when IT systems fail**?’. They also stressed the need for user-friendly websites. Those that had experiences of EMR talked about how little information there was available and the need for improved accessibility to medical information such as lab results, investigations and other relevant medical information.

The main issue that the participants talked about was that there was ‘**Poor communication between health care organizations’ IT systems**’. As no organization was fully updated with all the information, the participants expressed ‘**disappointment over poor IT systems**’ thus leading to low confidence in the EMR, which in turn led to a greater mistrust in e-health solutions

When I was at the hospital…they didn’t know what’s going on here at the PHCC…’Oh, I said, can’t you check on the computer?’ ‘No, they said, we don’t have access there…’…and then they asked about medicines and stuff like that…It should be accessible for them, otherwise I could sit there and talk a lot but maybe missing something…(Interview no 1, person no 1).

The issue of **‘Mistrust in e-health from health care organizations**’ was also raised. One of the participants had relatives working in a healthcare organization, and was told that they did not want digital tools to be too successful as this might lead to higher accessibility and a potential risk for work overload.

As I’ve been told, you´ve been against it before, you would be overloaded with phone calls when people would start reading the lab results on line. Have you experienced anything like this yet? Because my daughter worked here as a nurse, and I asked her why you don´t put the lab results online….and she told me that you wouldn´t be able to do anything else but answer the phone in that case… (Interview no 2, person no 5)

### ‘*Wanting and needing to move forward’*

This category revealed interest among the participants for e-health solutions. Several participants expressed that there was no possibility to turn back when it came to new technology and that the future was inevitable. These attitudes were compiled under the sub-category **‘Cannot stop development’**. Patients having positive experiences of digital tools were generally more interested in learning new technology and came with suggestions about improving or developing the services, for example making it possible to track referrals to hospital clinics or making lab results available for patients in the EMR online. Some of the patients expressed ‘**Curiosity and interest for digital tools and technical solutions’** as self-monitoring of measurements as blood pressure, blood sugar levels or thumb ECG. Receiving adequate and tailored information for the elderly as well as counselling about how to find and use the digital tools was described as crucial for adopting such instruments. Thus, the participants stressed the **‘Need for help and information concerning e-health’.**

Several of the respondents had developed learning strategies to overcome difficulties with the new technology **(‘To learn on older days’**), for example by attending courses or by getting help from younger family members, as one participant expressed it:

…so when I got this new phone (smartphone)…when you are so stupid as I was…how do you call?…I couldn’t figure out how to call…and you don't get a little brochure about how to do it, I was supposed to download a document with many A4 pages for printing, and I couldn't do it. So I went and asked those who knew, the younger ones. (Interview no 2, person no 1)

### ‘Concerns to be addressed for making e-health a good solution’

Talking about the future, participants expressed fear about reduced accessibility to health care for the elderly caused by **‘Lack of triage with online booking’** if younger patients overuse the system. Other risks that they talked about were increased costs for both the individuals and distribution of costs in the health care system with a fear that technology might replace human contact and put further pressure on the health care personnel. These concerns were compiled in the subcategory **‘Accessibility, costs and other risks with e-health’**

The development has gone wrong, to put all the money on machines and applications and computers…The human contact is important…and that there is enough personnel no matter what, to take care of the job and then go home and rest… (Interview no 3, person no 3)

The participants were also concerned about **‘Lack of time for physicians despite e-health’**:

He (the doctor) doesn’t have more time on the phone or on the screen, he is busy anyway regardless…’ ((Interview no 1, person no 3)

There was also a concern for lack of consideration for those not interested in using digital tools, who would be discriminated, and also **‘Insecurity with e-health in emergency situations’**:

If there is an emergency, it doesn’t go faster because I have an application…I might have to get in contact with somebody there, and perhaps the doctor there cannot assess me and sends me to the emergency room anyway…I don’t think an app should take that decision…’ (Interview no 3, person no 1)‘**Potential advantages with e-health versus ordinary health care**

Patients were able to see e-health as an improvement, an alternative or a complement to the existing health care system and some of them expressed beliefs about **‘Better access with digital consultations’**:

I had a rash on my leg once…and waited till the day after, which was a Saturday, and then had to go in…she (the doctor) said that it was herpes as soon as I went in… and then I had to rush to the pharmacy, you only have 72 hours. I could have showed it sooner on a screen, instead I had to call and wait in the phone queue…(Interview no 1, person no 5)

The drug list from the pharmacy, on paper and not always updated, was most used by the participants. Having digital access to information about the medication was described as another potential advantage, as in the subcategory: **‘Practical and safe with a comprehensive drug list in the mobile’**:

Yes, it would have been good to have it (the drug list) in, without having any papers, I think so. In my mobile phone. (Interview no 2, person no 1).

They talked about joint IT systems in terms of: **‘E-health a future way to reduce bureaucracy, demands and time’** and that it would be good to be able to book appointments online instead of being stuck in a telephone queue that was only accessible at certain hours, describing **‘Online booking as a complement’**. However, some participants expressed that e-health is not always necessary in urban areas but could have advantages in distant geographical areas or in certain situations, as compiled in the sub-category **‘Advantages with digital tools for some’.**

But maybe…it would work perfectly well to see him (the GP) through the computer…when I get allergy in the spring, I've had it more than once…or if my eyes get swollen from make-up or something…then I can see myself sitting and talking to him through the computer… (Interview no 2, person no 3)

### ‘Need for speed, access and correct comprehensive information’

The participants had **‘Expectations of higher accessibility with e-health’** and stressed especially the **‘Need for fast e-health accessibility in emergency situations’**, and also the need for a comprehensive and accessible EMR including relevant information such as lab results and an updated drug-list, as described in the subcategories **‘Importance of trustworthy information online’**, and **‘Expectations of lab results online’**.

Some of the participants expressed the **‘Need for video consultations in certain situations’**, for example to avoid sitting in a full waiting-room during flu season, outside office hours, for short contacts or when feeling insecure about the need for a doctor consultation.

Something else I think about when you mention it (video consultation) is that if I have something, but I am not sure what it might be, and I manage to get in contact with him (the GP) and show him a photo, maybe he could assess what my problem was… (Interview no 1, person no 6).

The need for updated information about drugs was described in the subcategory **‘Need for comprehensive drug list’.**

I have always a note with my medication in my wallet, for emergency cases…and definitely want to have digital access to one, on my phone for example… (Interview no 1, person no3)

## Discussion

### Main findings

Eight categories were derived from 36 subcategories and resulted in the main theme ‘The elderly’s ambivalence towards e-health: reluctant curiosity, a wish to join and need for information and learning support’. There were participants that reported low experience of digital tools and different levels of knowledge about how to access health care online. The participants with some experience of e-health described unmet expectations, expressed fear for reduced accessibility to health care and increased costs with e-health and the need for a comprehensive and accessible EMR. Some of the elderly patients expressed mistrust in their own capabilities, describing a generation gap with younger people being more accustomed to the new technology. In general, the patients reported trust and satisfaction with the usual care at the PHCC. However, they stressed the need for higher accessibility to health care outside office hours. Video consultations, text messages or digital reminders as a cognitive support were regarded as a positive complement to usual care or in certain situations.

### Strengths of this study

This study is, to our knowledge, the first to explore the attitudes of elderly people in Sweden towards e-health in focus groups and, in a broader perspective, in a time when fast technology development and high degree of digitalization in the population [19] might facilitate new forms of organizing and financing health care. The method allowed participants to express personal experiences and opinions. An advantage of focus group interviews is that the debate within the group could facilitate expression of attitudes and expectations that might have been left undeveloped in an individual in-depth interview. All three focus group interviews were judged to have had rich discussions, rather than individual opinions. This is considered as a strength in this study.

The patients in our study did not have any previous acquaintance with the interviewers or with each other, thus minimizing the risk for bias according to a realistic point of view [20]. The interviewed patients attended focus group interviews at three PHCCs in different socioeconomic areas, and we believe this to be a strength of the study by taking into consideration that socioeconomics influences access to and use of internet and digital tools, and therefore attitudes related to them, hence increasing the study’s credibility. Another strength of this study is that the participants were both males and females of different ages and had different levels of education and social background, which further increases credibility. The researcher (VMN), who performed the interviews, had no previous contact with the patients. During the interviews, one or two second researchers (BBB, SG, SC and MW) were present, in order to collect non-verbal communication or ask complementary and clarifying questions at the end of the interview, in order to further increase the credibility of the study. The co-interviewers participated in the process of including patients in the study on the PHCC they worked at, however, not being present at the interviews at the same PHCC. A further strength of the study is that neither the main interviewer nor the co-interviewers had previous relation to or contact with the patients. Another strength of the study is the investigator triangulation and the use of a known methodology used during data analysis, which also increases the reliability and credibility of the results. The participants were asked during the interviews whether their statements had been correctly perceived, with the interviewer making summaries of the narrative throughout the interview period, in order to validate the results.

### Limitations of this study

Four to six participants are recommended for focus groups and we managed to include a total 15 patients with 6, 6 and 3 patients in the groups respectively, due to low attendance at one of the interviews. The smallest group represented a PHCC in an area with lower socioeconomic status, which might have influenced the attendance to the interview. The risk of small focus groups is limited discussions. However, despite having only three participants there were rich discussions also in this group that was heterogeneous in both gender, education level and age variation. The limited number of the participants in this group might however be a weakness of the study, given the fact that this interview was made with patients of lower socioeconomic status.

The three participants in group no 3 were younger than the participants in other groups (65, 66, 80 years old). Despite the age difference, the younger participants (65 and 66) had less positive attitudes and experiences of digital tools than the 80-year-old participant, but also lower educational level, which indicated that interest in technology might not necessarily be related to age. This focus group interview with three participants included rich material and extensive discussions. Together with the interesting dynamics that occurred within the group due to different attitudes and experiences and the collection of new information, we decided to use the material for the analysis. After three focus group interviews, the material was judged to contain sufficient information for analysis. Similar findings were found in all focus groups interviews, and we therefore decided not to perform additional interviews.

The researcher who performed the interviews (VMN) worked as a GP. She explained her role as a researcher in the beginning of the interviews and did not address any questions related to her pre-understanding of the studied topic or the cultural context. However, her pre-understanding might have influenced her objective reflexivity and this may have been a limitation of the study.

### General discussion of the results

The findings support Rogers’ ‘Diffusion of innovations theory’ stating that different behaviors (‘early adopters’, ‘early majority’, ‘late adopters’, ‘late majority’, ‘laggards’) depend on the perceived advantages or the degree of difficulty with the innovation [14]. Patients expressing lack of interest for digital tools were generally less accustomed to using the Internet or smartphones, some of them declared a strong aversion to technology and feeling secure with the follow-up system including regular visits at the PHCC. These beliefs seemed to be based on low or zero experience of digital tools. Correspondingly, patients in our study that were more positive and curious about potential benefits of e-health often had some experience of digital tools.

Some of the participants in this study described clear advantages with video-based appointments in certain situations. However, they also described a feeling of insecurity and skepticism. This is consistent with findings in a qualitative study of patients’ perceptions of video-based consultations, which showed that patients appreciated the enhanced convenience, reduced costs and improved associated punctuality, but also expressed a sense of alienation arising from the use of technology, and problems with doctor-patient communication [21]. The same conclusion arose from a patient survey on perceptions of video-based appointments thus indicating that most patients are likely to be accepting of video calls and that most have the required technology [22]. However, there are still questions about how to successfully implement this adaptation of e-health in the health care practice [20]. The patients in our study saw clear advantages with e-health as a complement but not a substitute to usual care, consistent with a British study finding that acceptance of technology among the elderly is greater when not perceived to be replacing in-person care [23].

The patients in this study reported mistrust in IT systems in health care, which they described as poor. The participants in all three focus groups were unanimous in their beliefs about the barrier created by lack of interoperability between EMRs at different health care providers and its consequences. Considering the fast development and spread of IT solutions in other areas, participants were puzzled and frustrated over policy makers’ inertia in this matter and expressed a strong need for user-friendly and accessible digital tools, as well as for learning support and information about them. This is one of the most important findings, as trust in the provider of the tool and the compatibility with patients’ needs are important factors for patients’ adherence to e-health services, according to a study done with in-depth interviews in Sweden [24]. Another Swedish study indicated that the most significant factor for patients’ adoption of e-health care systems is the system's ability to support autonomy in everyday life and that customization is crucial [25]. The participants in our study reported a large diversity of attitudes, expectations and needs of e-health solutions. The results indicate that digital tools in health care should be tailored to fit different behaviors among patients and marketed with different strategies in order to ensure successful implementation.

The interviewed participants in this study expressed both positive and negative attitudes towards tele-monitoring of chronic disease, results consistent with findings in a recent meta-synthesis of qualitative studies upon how users (both staff and patients) perceive the technology of telehealth in management of COPD, concluding that future research need to include potential users at an earlier stage of service development [26]. None of the participants in this study had extensive experience of monitoring chronical illnesses over time using e-health. More evidence in this area is therefore needed, as a recent systematic review concluded that even if interactive mobile-phone interventions may provide benefit in supporting long-term illnesses, there are significant information gaps such as acceptability or long-time effect [8]. Furthermore, novel technologies will be accepted by the elderly population if health information that is secure, readily accessible and easy to understand is provided, as concluded in a Canadian qualitative study [27].

The participants’ heterogeneity of reported attitudes and beliefs in the present study is consistent with findings in a recent analysis made by the Swedish association of local authorities and regions (SALAR) [28] exploring individuals’ attitudes towards health care in general. The report concludes that different personality types, values, lifestyles, attitudes and behavior in the population need different targeted health care interventions [28]. According to this report, the Swedish health care system today has been designed and probably adjusted to people who see themselves as competent and are able to navigate the health care system, part of them being more anxious and needing fast access to health care for reassurance and comfort. A total of 25% percent of this study population [28] was between 65–80 years old, matching the population’s characteristics in our study. Given the fast development of technology during recent years [3], it is expected that this group of elderly will, with the right support, eventually accomplish higher confidence in digital tools use as the societal development moves further. However, it is important to also address the needs of patients with less confidence in themselves and the health care system and with aversion to technology. To be able to seek, find, understand, appraise, and apply health information from electronic resources or to solve or address a health problem has been defined as e-health literacy [29]. One way to increase e-health literacy has been reported to be interventions using short e-learning courses (two weeks with 10 min sessions each day) [30]. This kind of intervention has also found to be effective in elderly people [31], which also reported that collaborative learning did not differ from individualistic learning. Other studies have revealed that the elderly population may successfully adopt new technologies with appropriate support [32,33]. When implementing innovative e-health interventions potential e-health literacy must be taken into consideration, and interventions should address elderly patients’ different attitudes, beliefs, behavioral patterns and needs for information and learning support.

## Conclusions

The Swedish elderly patients in this study described ambivalence towards e-health, raising concerns as accessibility to health care, mistrust in poor IT systems or impaired abilities to cope with technology. They also expressed a wish and need to move forward albeit with reluctant curiosity. Successful implementation of e-health interventions should be tailored to target different attitudes and needs with strong focus on information and support for the elderly.

## References

[1] Statistics Sweden [cited 2017 Jul 5]. Available from: http://www.scb.se/en/finding-statistics/statistics-by-subject-area/population/population-projections/population-projections/pong/publications/the-future-population-of-sweden-2017-2016

[2] Johansson SE, Midlov P, Sundquist J, et al. Longitudinal trends in good self-rated health: effects of age and birth cohort in a 25-year follow-up study in Sweden. Int J Public Health. 2015;60:363–373. 25650292 10.1007/s00038-015-0658-y

[3] Ekholm A, Jebari K, Nilsson F, et al. Bortom IT. Om hälsa i en digital tid. [Beyond IT. About Health in a Digital Time] Institutet För Framtidsstudier; 2016 [cited 2018 Jul 1]. Available from: https://www.iffs.se/publikationer/if-rapporter/bortom-it-om-halsa-i-en-digital-tid/

[4] Krishna S, Boren SA, Balas EA Healthcare via cell phones: a systematic review. Telemed J E Health. 2009;15:231–240. 19382860 10.1089/tmj.2008.0099

[5] Lin H, Wu X Intervention strategies for improving patient adherence to follow-up in the era of mobile information technology: a systematic review and meta-analysis. PLoS One. 2014;9:e104266. 25100267 10.1371/journal.pone.0104266 PMC4123963

[6] Williams V, Price J, Hardinge M, et al. Using a mobile health application to support self-management in COPD: a qualitative study. Br J Gen Pract. 2014;64:e392–e400. 24982491 10.3399/bjgp14X680473 PMC4073724

[7] Bengtsson U, Kjellgren K, Hallberg I, et al. Improved blood pressure control using an interactive mobile phone support system. J Clin Hypertens. 2016;18:101–108. 10.1111/jch.12682 PMC5057328 26456490

[8] McBain H, Shipley M, Newman S The impact of self-monitoring in chronic illness on healthcare utilisation: a systematic review of reviews. BMC Health Serv Res. 2015;15:565. 26684011 10.1186/s12913-015-1221-5 PMC4683734

[9] Xiang R, Li L, Liu SX Meta-analysis and meta-regression of telehealth programmes for patients with chronic heart failure. J Telemed Telecare. 2013;19:249–259. 24163234 10.1177/1357633X13495490

[10] Polisena J, Tran K, Cimon K, et al. Home telemonitoring for congestive heart failure: a systematic review and meta-analysis. J Telemed Telecare. 2010;16:68–76. 20008054 10.1258/jtt.2009.090406

[11] Frydenberg K, Brekke M Poor communication on patients’ medication across health care levels leads to potentially harmful medication errors. Scand J Prim Health Care. 2012;30:234–240. 23050954 10.3109/02813432.2012.712021 PMC3520418

[12] Tully MP, Kettis A, Hoglund AT, et al. Transfer of data or re-creation of knowledge – experiences of a shared electronic patient medical records system. Res Social Adm Pharm. 2013;9:965–974. 23562042 10.1016/j.sapharm.2013.02.004

[13] Ajzen I The theory of planned behavior. Organ Behav Hum Decis Process. 1991;50:179–212.

[14] Rogers EM Lessons for guidelines from the diffusion of innovations. Joint Comm J Qual Improv. 1995;21:324–328. 10.1016/s1070-3241(16)30155-9 7581733

[15] Bujnowska-Fedak MM, Pirogowicz I Support for e-health services among elderly primary care patients. Telemed J E Health. 2014;20:696–704. 24359252 10.1089/tmj.2013.0318 PMC4106384

[16] Kitzinger J Qualitative research. Introducing focus groups. BMJ. 1995;311:299–302. 7633241 10.1136/bmj.311.7000.299 PMC2550365

[17] Graneheim UH, Lundman B Qualitative content analysis in nursing research: concepts, procedures and measures to achieve trustworthiness. Nurse Educ Today. 2004;24:105–112. 14769454 10.1016/j.nedt.2003.10.001

[18] Burnard P, Gill P, Stewart K, et al. Analysing and presenting qualitative data. Br Dent J. 2008;204:429–432. 18438371 10.1038/sj.bdj.2008.292

[19] Davidsson P Svenskarna och Internet. Undersökning om svenskarnas internetvanor. Stockholm; 2016 Available from: https://2018.svenskarnaochinternet.se/

[20] Krueger RA Analyzing focus group interviews. J Wound Ostomy Continence Nurs. 2006;33:478–481. 17133134 10.1097/00152192-200609000-00004

[21] Harrison R, Macfarlane A, Murray E, et al. Patients’ perceptions of joint teleconsultations: a qualitative evaluation. Health Expect. 2006;9:81–90. 16436164 10.1111/j.1369-7625.2006.00368.x PMC5060326

[22] Gardner MR, Jenkins SM, O’Neil DA, et al. Perceptions of video-based appointments from the patient’s home: a patient survey. Telemed J E Health. 2015;21:281–285. 25166260 10.1089/tmj.2014.0037 PMC4378342

[23] Currie M, Philip LJ, Roberts A Attitudes towards the use and acceptance of eHealth technologies: a case study of older adults living with chronic pain and implications for rural healthcare. BMC Health Serv Res. 2015;15:162. 25888988 10.1186/s12913-015-0825-0 PMC4415301

[24] Jung ML, Loria K Acceptance of Swedish e-health services. J Multidiscip Healthc. 2010;3:55–63. 21289860 10.2147/JMDH.S9159 PMC3024889

[25] Forsberg A, Ostlund B Elderly people’s perceptions of a telehealthcare system: relative advantage, compatibility, complexity and observability. J Tech Hum Serv. 2013;31:218–237.

[26] Brunton L, Bower P, Sanders C The contradictions of telehealth user experience in chronic obstructive pulmonary disease (COPD): a qualitative meta-synthesis. PLoS One. 2015;10:e0139561. 26465333 10.1371/journal.pone.0139561 PMC4605508

[27] Ware P, Bartlett SJ, Pare G, et al. Using eHealth technologies: interests, preferences, and concerns of older adults. Interact J Med Res. 2017;6:e3. 28336506 10.2196/ijmr.4447 PMC5383803

[28] SALAR 2016; [cited 2017 Jul 5]. Available from: https://skl.se/halsasjukvard/kunskapsstodvardochbehandling/primarvardutveckling/primarvardeninnovativaarbetssatt.8801.html

[29] Norman CD, Skinner HA eHealth literacy: essential skills for consumer health in a networked world. J Med Internet Res. 2006;8:e9. 16867972 10.2196/jmir.8.2.e9 PMC1550701

[30] Mitsuhashi T Effects of two-week e-learning on eHealth literacy: a randomized controlled trial of Japanese internet users. PeerJ. 2018;6:e5251. 30013857 10.7717/peerj.5251 PMC6047505

[31] Xie B Effects of an eHealth literacy intervention for older adults. J Med Internet Res. 2011;13:e90 22052161 10.2196/jmir.1880 PMC3222191

[32] Luijkx K, Peek S, Wouters E “Grandma, you should do it-it's cool” older adults and the role of family members in their acceptance of technology. Int J Environ Res Public Health. 2015;12:15470–15485. 26690188 10.3390/ijerph121214999 PMC4690935

[33] Tan CC, Cheng KK, Wang W Self-care management programme for older adults with diabetes: an integrative literature review. Int J Nurs Pract. 2015;21: 115–124. 26125579 10.1111/ijn.12388

